# Large-scale performance assessment of the BG-Counter 2 used with two different mosquito traps

**DOI:** 10.1186/s13071-024-06338-x

**Published:** 2024-06-27

**Authors:** Leif Rauhöft, Tatiana Șuleșco, Sara M. Martins Afonso, Jonas Schmidt-Chanasit, Hanna Jöst, Felix G. Sauer, Renke Lühken

**Affiliations:** 1 Bernhard Nocht Institute for Tropical Medicine, Hamburg, Germany; 2 Faculty of Mathematics, Informatics and Natural Sciences, Universität Hamburg, 22609 Hamburg, Germany

**Keywords:** Mosquito trap, Automatic counting, Accuracy, Culex, CO_2_-trap, Gravid trap

## Abstract

**Background:**

Mosquitoes are important vectors of pathogens. They are usually collected with CO_2_-baited traps and subsequently identified by morphology. This procedure is very time-consuming. Automatic counting traps could facilitate timely evaluation of the local risk for mosquito-borne pathogen transmission or decision-making on vector control measures, but the counting accuracy of such devices has rarely been validated in the field.

**Methods:**

The Biogents (BG)-Counter 2 automatically counts mosquitoes by discriminating the size of captured objects directly in the field and transmits the data to a cloud server. To assess the accuracy of this counting device, 27 traps were placed at 19 sampling sites across Germany and used in daily, weekly or bimonthly intervals from April until October 2021. The BG-Counter 2 was attached to a CO_2_-trap (BG-Pro trap = CO_2_-Pro) and the same trap was converted to also attract gravid mosquitoes (upside-down BG-Pro trap with a water container beneath = CO_2_-Pro-gravid). All captured mosquitoes were identified by morphology. The number of females (unfed and gravid), mosquito diversity and the number of identified specimens in relation to the counting data of the BG-Counter were compared between the two trapping devices to evaluate sampling success and counting accuracy.

**Results:**

In total 26,714 mosquitoes were collected during 854 trap days. The CO_2_-Pro-gravid trap captured significantly more mosquitoes per trap day for all specimens, gravid females and non-gravid females, while there was no difference in the mosquito diversity. The linear model with the captured mosquitoes as a response and the counted specimens as a predictor explained only a small degree of the variation within the data (*R*^2^ = 0.16), but per individual trap the value could reach up to 0.62 (mean *R*^2^ = 0.23). The counting accuracy for the daily samples had a significant positive correlation with sample size, resulting in higher accuracy for the CO_2_-Pro-gravid trap and higher accuracy for sites and sampling months with high mosquito abundance.

**Conclusions:**

While the accuracy of the BG-Counter 2 is quite low, the device is able to depict mosquito phenology and provide information about local population dynamics.

**Graphical Abstract:**

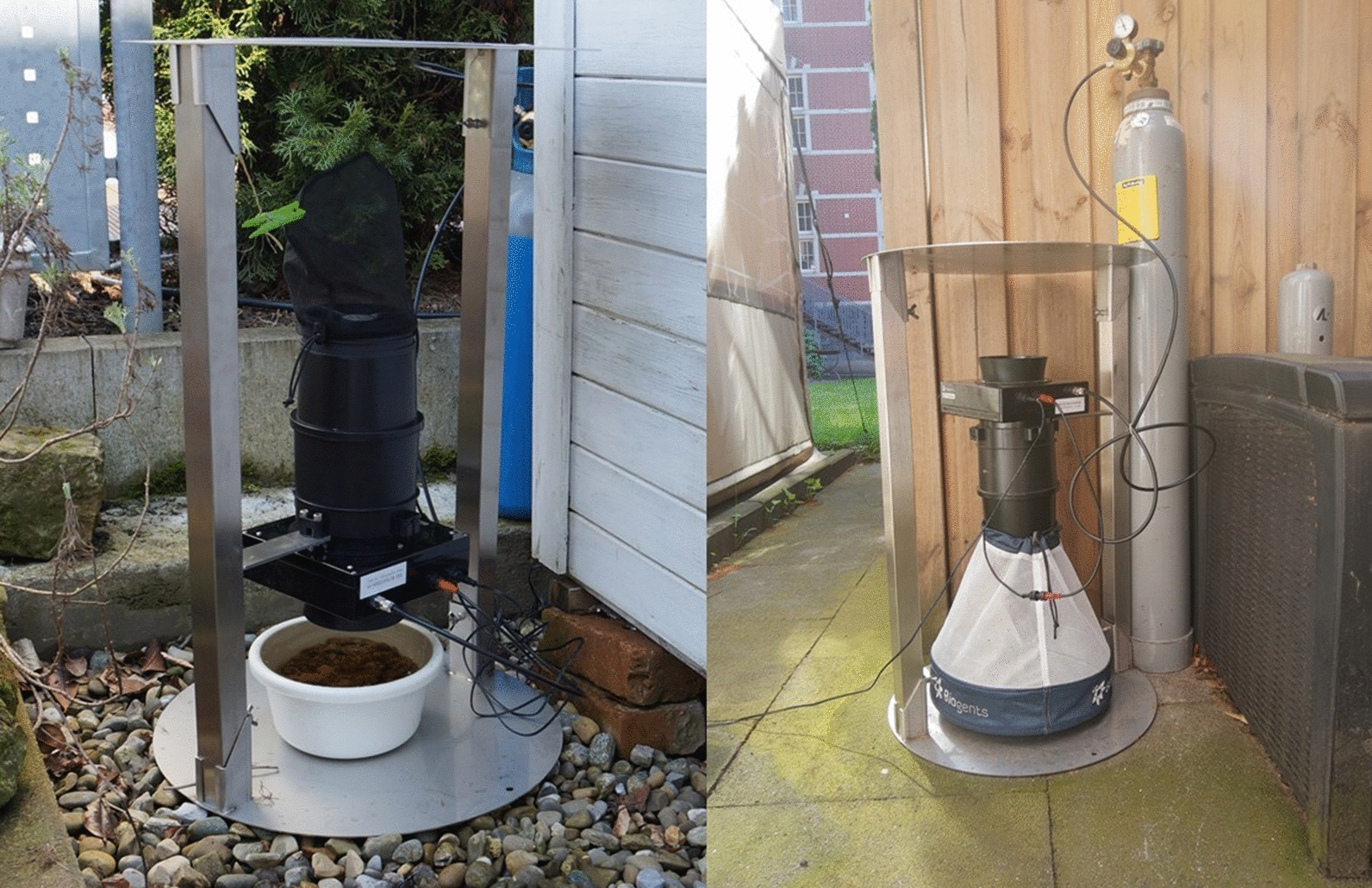

**Supplementary Information:**

The online version contains supplementary material available at 10.1186/s13071-024-06338-x.

## Background

Mosquitoes are important vectors of pathogens, with malaria parasites and dengue virus having the highest relevance on a global scale. It is estimated that these two pathogens alone result in more than 500 million annual cases worldwide, especially in tropical and subtropical regions [1, 2]. However, due to climate warming in particular, there are also emerging mosquito-borne pathogens in Central Europe such as the West Nile virus and the Usutu virus [3, 4]. Since the first emergence of Usutu virus (2011/2012) and West Nile virus (2018) in Germany, both viruses have been regularly detected in mosquitoes, horses, birds and humans [5, 6].

Mosquito monitoring and surveillance programs provide the foundation for identifying the spatial–temporal risk of mosquito-borne pathogen transmission. In these programs, mosquitoes are usually collected with CO_2_-traps in the field and subsequently identified and screened in the laboratory. This process results in a significant delay between the collection and the subsequent identification and pathogen screening of mosquitoes. Automatic traps for counting mosquitoes could significantly reduce the time lag, at least providing mosquito data in near-real time directly from the field. These readily available data could, for instance, enable the rapid evaluation of the success of vector control measures, i.e. measuring the abundance pre- and post-treatment, or inform us about the best time of the day for adulticide spraying. Furthermore, the data could be used to estimate the local pathogen transmission risk.

There are a few promising automatic counting devices for mosquitoes. These include different prototypes developed by Chen et al. [7], Lai et al. [8] and Gonzales-Perez et al. [9]. Currently, the BG-Counter (Biogents, Regensburg, Germany) is the only commercially available automatic mosquito counting system. Besides the BG-Counter, the prototypes described Lai et al. [8] and Gonzales-Perez et al. [10] are the only ones with published data from the field. For the prototype reported by Lai et al., mosquitoes were collected at two sites in China from May to August 2021. The average daily counting accuracy was 79.4% in an open field and 64.9% near a residence. For the prototype reported by Gonzales-Perez et al., mosquitoes were collected at two sampling sites in Spain—at site 1 from July to October 2021, with an average daily counting accuracy of 89.1%, and at site 2 from June until September 2022, with an average daily counting accuracy of 88.1%. Additionally, this prototype is able to distinguish between the genera *Aedes* and *Culex.* The BG-Counter has been used for a wider range of applications. For instance, it was used to assess the efficacy of insecticide barrier treatments in remote areas as shown for an inhabited island in a marine bay in Australia [11] or in more urban settings as in Illinois, USA [12]. Another example is the evaluation of the impact of a hurricane on the mosquito populations of an inhabited island in the USA [13] or the assessment of the dispersal of mosquitoes from highly productive breeding sites on bay islands to the mainland of Australia [14]. The most comprehensive evaluation of the BG-Counter took place in North Carolina, USA [15]. Five BG-Counters were placed in five different counties, where the mean daily accuracy ranged from 9.4 to 80.1%. Linear regressions between the BG-Counter and actual mosquito counts resulted in correlation coefficients ranging from 0.0085 to 0.95 depending on the sampling site. However, so far, there are no evaluations of the performance of BG-Counters for Europe.

The study presented herein is the first large-scale systematic evaluation of the BG-Counter, using 27 BG-Counters deployed over 19 sampling sites in Germany over one complete field season. We aimed to analyse the counting accuracy of the BG-Counter 2 when using it as a standard version with a CO_2_-lured BG-Pro or a CO_2_-lured BG-Pro combined with a gravid trap. We compared the trapping efficiency of the two trap versions for the total number of mosquitoes, blood-fed and gravid specimens, and species diversity. In addition, we analysed the counting accuracy of both trap versions depending on the sampling site, time period and sample size.

## Methods

Similar to the other automatic counting devices, the BG-Counter uses infrared light-emitting diodes and light detectors to measure the obstruction of light caused by a passing insect and thereby discriminate mosquitoes from other objects [16]. The signal detected by the light sensors is dependent on the size and wingbeat frequency of the insects. Additionally, the BG-Counter is fitted with sensors for temperature, relative humidity and ambient light. By using a 4G cellular communication module and a SIM card, it can transmit all data to an online server every 15 min. The BG-Counter can also be controlled remotely, i.e. the fan can be switched on or off and the CO_2_ outlet can be opened or closed.

**Fig. 1:**
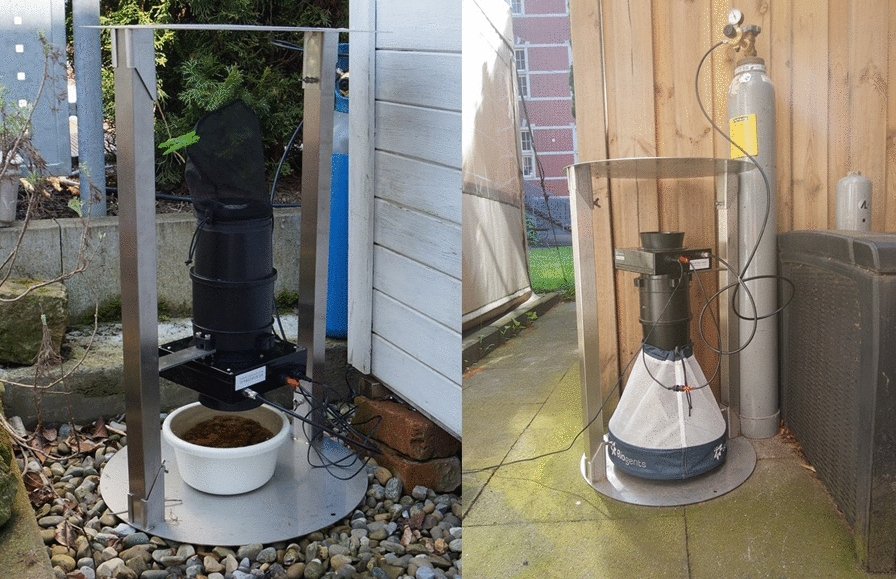
Trap versions: CO_2_-Pro-gravid (left) and CO_2_-Pro (right), each equipped with a BG-Counter 2 in combination with the BG-Pro and BG-Trap-Station

Two different traps were used with the BG-Counter (Fig. 1): the standard version, as suggested in the BG-Counter user manual, consisting of the BG-Pro, BG-Counter 2 and BG-Trap Station (CO_2_-Pro), and a second version converting the standard version into a combination of a CO_2_ and gravid trap (CO_2_-Pro-gravid). For the gravid version, the entire trap was turned 180°. Beneath the gravid trap, a water container (2.6 l) is placed with approximately 60 g of hay pellets and a tablet containing toxins of *Bacillus thuringiensis israelensis* (Culinex Tab plus, Becker GmbH, Ludwigshafen am Rhein, Germany), preventing the development of mosquitoes. For the CO_2_-Pro-gravid version, the fabric trap body is not attached since the catch bag otherwise would be squashed by its weight (Fig. 1). Both the standard and CO_2_-Pro-gravid version were used with the adjustable pressure regulator set to 1.5 kg/day. As a technical note, the BG-Counter cannot simply be turned around, since the sensor openings would be exposed to rain, allowing the entry of moisture (Additional file 1: Fig. S1). The entering funnel has to be replaced by a thread adapter enabling the connection of the BG-Counter to the BG-Pro trap in an upside-down position. For this purpose, new holes were drilled into the thread adapter and countersunk, since the screw heads would prevent the mounting to the trap.

**Fig. 2:**
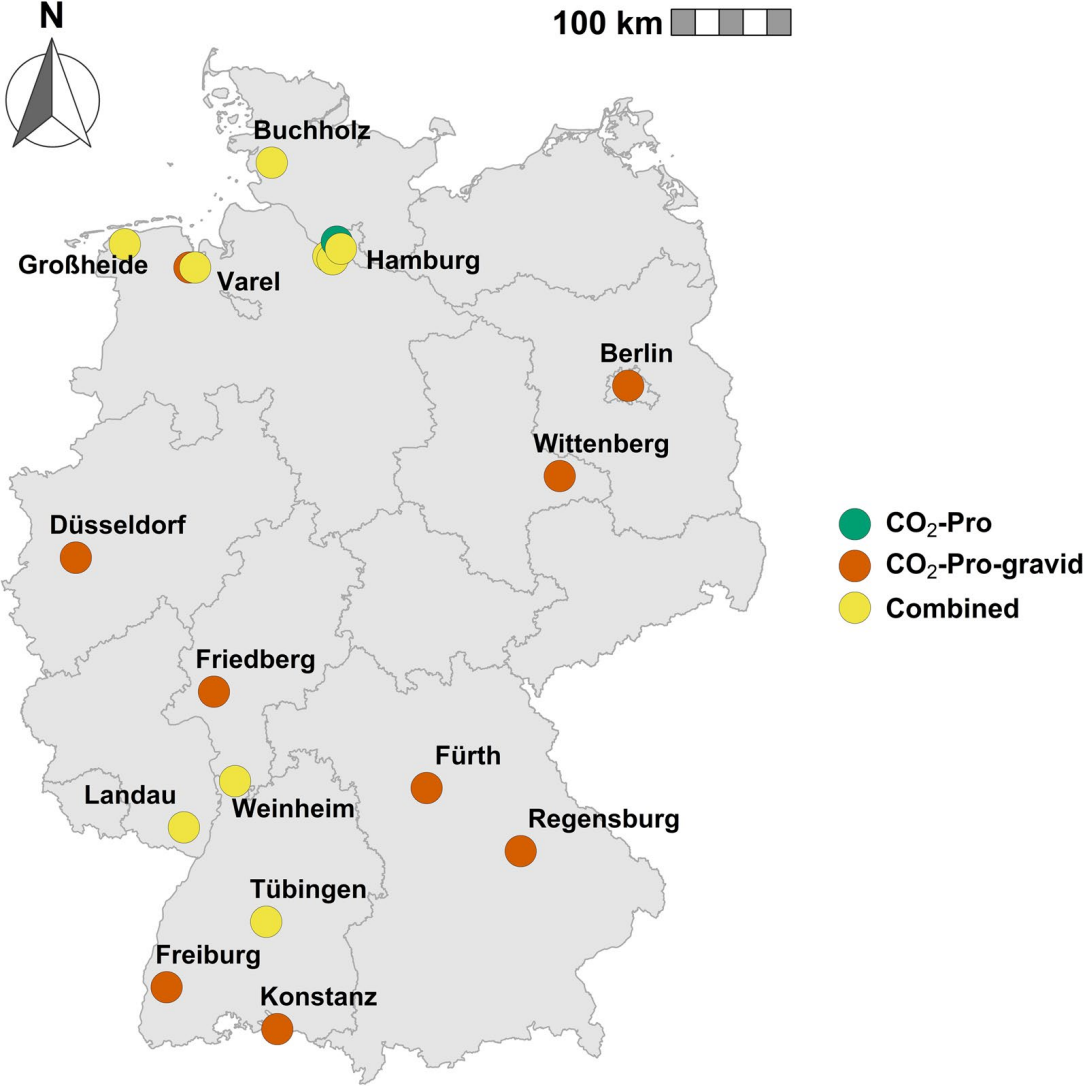
Map of sampling sites in Germany

In 2021, mosquitoes were collected at 19 different sampling sites in Germany (Fig. 2). A total of 27 traps were deployed, comprising 10 CO_2_-Pro traps and 17 CO_2_-Pro-gravid traps. In order to compare the efficacy and accuracy of the two trap versions, we equipped eight sampling sites with both traps. Nine sites were exclusively equipped with the CO_2_-Pro-gravid version and two sites were equipped with the CO_2_-Pro trap version only. During each sampling event, the traps were running for approximately 24 h. A unique label was placed in the capturing net and preserved at −20 °C until further analysis. The sampling took place from April until October and was mainly conducted in private gardens in cooperation with volunteers. Six sampling sites were sampled as often as possible (nearly daily), five sampling sites were sampled weekly and eight on a biweekly basis. The distance between the two trap versions was not standardized but was chosen considering the available space, access to electrical sockets and the convenience of the voluntary helpers. It ranged between 5 and 20 m. At one site, the distance was shorter but the traps were separated by a garden shed. Thus, an interaction between the traps at the sampling sites for the trap comparison cannot be completely excluded. A 12 V portable freezer (Dometic CFX3 55, Dometic, Solna, Sweden) was used to maintain the cold chain during transport to the laboratory. All female mosquitoes were identified by morphological analysis, using a taxonomic key [17]. Additionally, the blood-fed status of the caught mosquitoes was assessed using the Sella score [18], categorizing mosquitoes as unfed (Sella score 1) or as blood-fed from freshly engorged to gravid (Sella score 2–7).

The BG-Counter data were downloaded as a CSV file and the same unique ID was generated as used for the capturing nets. The unique ID was then used to align the identification data with the BG-Counter data. All counted and identified mosquitoes were summarized per sampling event. Additionally, the counting accuracy was calculated using the formulas published by Day et al. [15]. In the case that the counter undercounted the mosquitoes, the formula was the automatic count divided by the manual count times 100. In case the BG-Counter overcounted the actual number of mosquitoes, the formula was the manual count divided by the automatic count times 100. In order to assess whether the BG-Counters transmitted data successfully, we compared the number of morphologically identified trap days with the trap days delivered by the BG-Counter. To analyse the differences in the trapping efficiency and counting accuracy between CO_2_-Pro and CO_2_-gravid traps, only the nine sampling sites equipped with both trap versions were considered, and only the trap days with corresponding data of both trap versions. Additionally, the mean accuracy, the mean number of caught mosquitoes and the mean number of mosquitoes caught with a Sella score above 1 was calculated and compared between the two trap versions. For each sampling event, the Shannon diversity index was calculated and statistically compared between the two trap versions using a Kruskal–Wallis test. This statistical analysis only considered specimens that were identified to the lowest taxonomic level possible by morphological means [17], i.e. specimens which were only identified to the genus or family level were not included in this analysis. Only for sampling sites with more than 10 sampling events per season, Spearman correlation coefficients and linear models were calculated for each trap and sampling site to statistically analyse the relationship between the manually identified and automatically counted mosquitoes. A categorical factor was generated, dividing all sample sizes into small (0–10 mosquitoes/24 h) and large (> 10 mosquitoes/24 h), since higher accuracy was observed during times of higher mosquito abundance. A linear model with all available data points was fitted with the identified mosquitoes as the response and BG-Counter counts as the predictor to evaluate the general correlation between the two. To resolve the cause of differences in accuracy, a binomial generalized linear mixed model (GLMM) was fitted with accuracy as the response and the number of identified mosquitoes and the trap version used as predictors. A second binomial GLMM was performed using the accuracy as a predictor and the month of capture as a response. Both mixed models included sampling sites as random factors. All computational analysis was performed in R (version 4.4) using the RStudio integrated development environment (IDE) (version 2024.4.0) [19]. Additionally, functions from the following packages were used for data preparation, visualization and analysis: rstatix [20], dplyr [21], sp [22], ggplot2 [23], ggdist [24], ggpubr [25], lubridate [26], tidyr [27], vegan [28], lme4 [29], sf [30], geodata [31] and scales [32].

## Results

In 2021, 26,714 mosquitoes were captured during a total of 854 trap days with both trap versions. The average success rate of data transmission of the 27 BG-Counters was 85.9% (Additional file: Table S1). While 17 had a 100.0% success rate, the success rate of the 10 remaining BG-Counters ranged from 17.6 to 93.3%. The mosquitoes belonged to at least 20 species of five genera (Table 1). *Culex pipiens* sensu stricto (s.s.)/*Culex torrentium* was the most abundant mosquito taxon with a total of 5782 specimens (54.9%) for the CO_2_-Pro trap and 12,282 specimens (75.9%) for the CO_2_-Pro-gravid trap, followed by *Aedes vexans* (CO_2_-Pro: 2693 specimens, 25.6%; CO_2_-Pro-gravid: 596 specimens, 3.7%). All other taxa were considerably less frequent (maximal 310 specimens, maximal 1.9%).

**Table 1:** Number of caught mosquito species and corresponding percentages in brackets for both used trap versions: CO2-Pro and CO2-Pro-gravid

Taxon	CO_2_-Pro	CO_2_-Pro-gravid
*Aedes albopictus*	0 (0)	116 (0.7)
*Ae. annulipes* group	45 (0.4)	11 (0.1)
*Ae. caspius*	4 (0)	16 (0.1)
*Ae. cinereus*	7 (0.1)	2 (0)
*Ae. communis*	1 (0)	0 (0)
*Ae. geniculatus*	5 (0)	3 (0)
*Ae. japonicus*	31 (0.3)	45 (0.3)
*Ae. punctor*	1 (0)	2 (0)
*Ae. rusticus*	12 (0.1)	10 (0.1)
*Ae.* species	36 (0.3)	97 (0.6)
*Ae. sticticus*	7 (0.1)	310 (1.9)
*Ae. vexans*	2693 (25.6)	596 (3.7)
*Anopheles claviger* s.s./*An. petragnani*	52 (0.5)	27 (0.2)
*An. maculipennis* s.l.	36 (0.3)	17 (0.1)
*An. plumbeus*	7 (0.1)	2 (0)
*An.* species	20 (0.2)	18 (0.1)
*Coquillettidia richiardii*	56 (0.5)	43 (0.3)
*Culiseta annulata*	166 (1.6)	242 (1.5)
*Cs. morsitans*	3 (0)	2 (0)
*Cs.* species	0 (0)	1 (0)
Culicidae	832 (7.9)	740 (4.6)
*Culex modestus*	8 (0.1)	5 (0)
*Cx. pipiens* s.s./*Cx. torrentium*	5782 (54.9)	12,282 (75.9)
*Cx.* species	588 (5.6)	1408 (8.7)
*Cx. territans*/*Cx. hortensis*	1 (0)	0 (0)
Unidentified males	130 (1.2)	196 (1.2)
Total	10,523	16,191

**Fig. 3:**
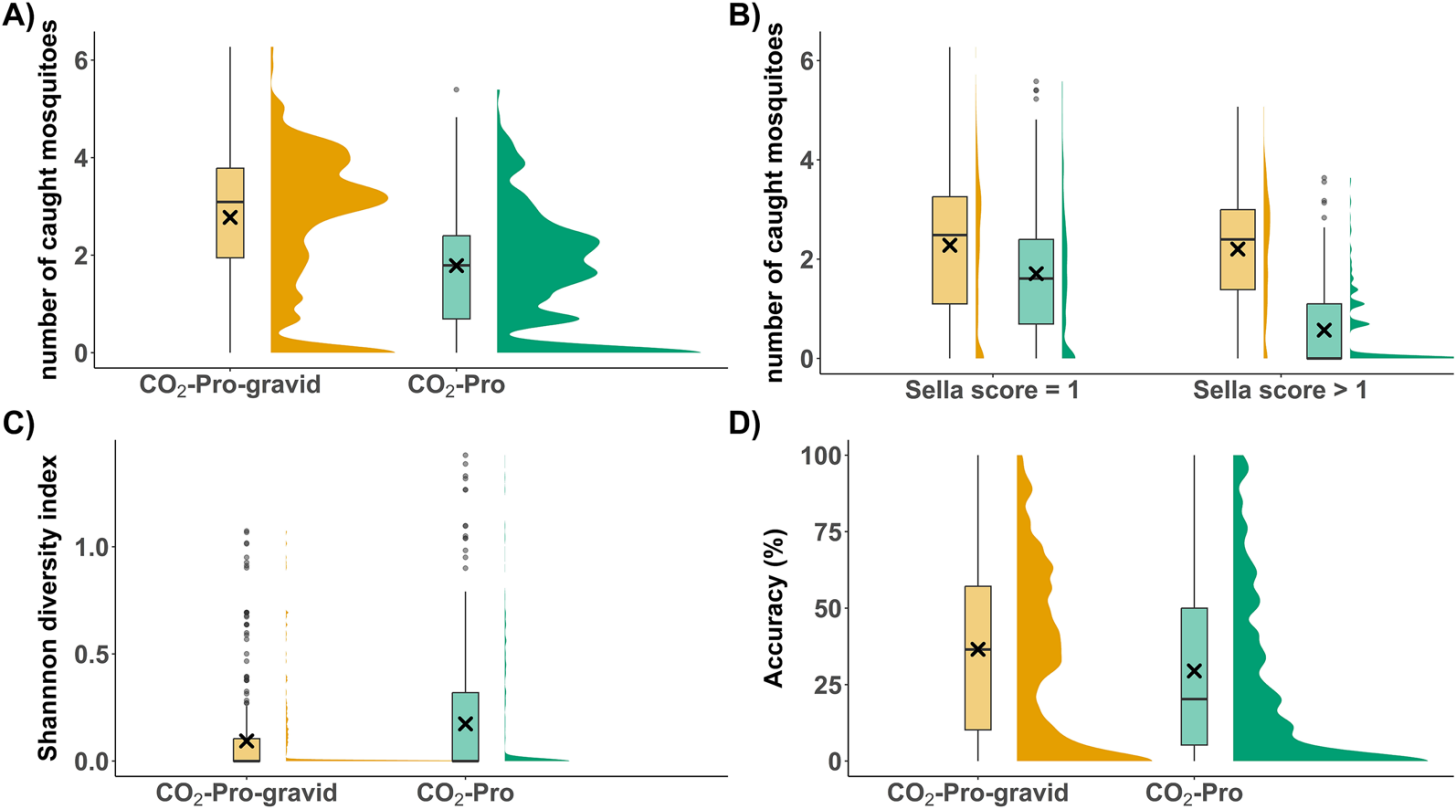
Trap version comparison with boxplots and half-density distributions: **A** number of caught mosquitoes, **B** captured mosquitoes with a Sella score of either 1 (unfed) or higher (blood-fed), **C** Shannon diversity index, **D** BG-Counter accuracy

The dataset for the trap comparison contains 283 trap days for each trap version. The BG-Counter generally overestimated the quantity of captured mosquitoes: 90.8% of the trap days for the CO_2_-Pro were overestimated and 91.2% for the CO_2_-Pro-gravid. For 8.1% of the trap days, the number of specimens was underestimated for both traps and accurate for 1.1% of trap days (CO_2_-Pro) and 0.7% (CO_2_-Pro-gravid). The CO_2_-Pro trap captured significantly fewer mosquitoes per trap day (mean number of specimens = 14.0, 95% confidence interval (95% CI) = 10.8–17.3 vs. 32.1, 95% CI = 27.0–37.2; Kruskal–Wallis, Chi-square = 68.7, *df* = 1, *P* < 0.0001; Fig. 3A), which was also true for mosquitoes with a Sella score of 1 (12.8, 95% CI = 9.6–16.0 vs. 21.7, 95% CI = 17.3–26.1 Kruskal–Wallis, Chi-square = 33.1, *df* = 1, *P* < 0.0001; Fig. 3B) or greater than 1 (1.7, 95% CI = 1.1–2.2 vs. 14.2, 95% CI = 12.0–16.4; Kruskal–Wallis, Chi-square = 210.9, *df* = 1, *P* < 0.0001; Fig. 3B). The difference in captured mosquito diversity per trap day was not significant for the Shannon diversity index (Kruskal–Wallis, Chi-square = 3.2, *df* = 1, *P* = 0.0738, Fig. 3C).

**Fig. 4:**
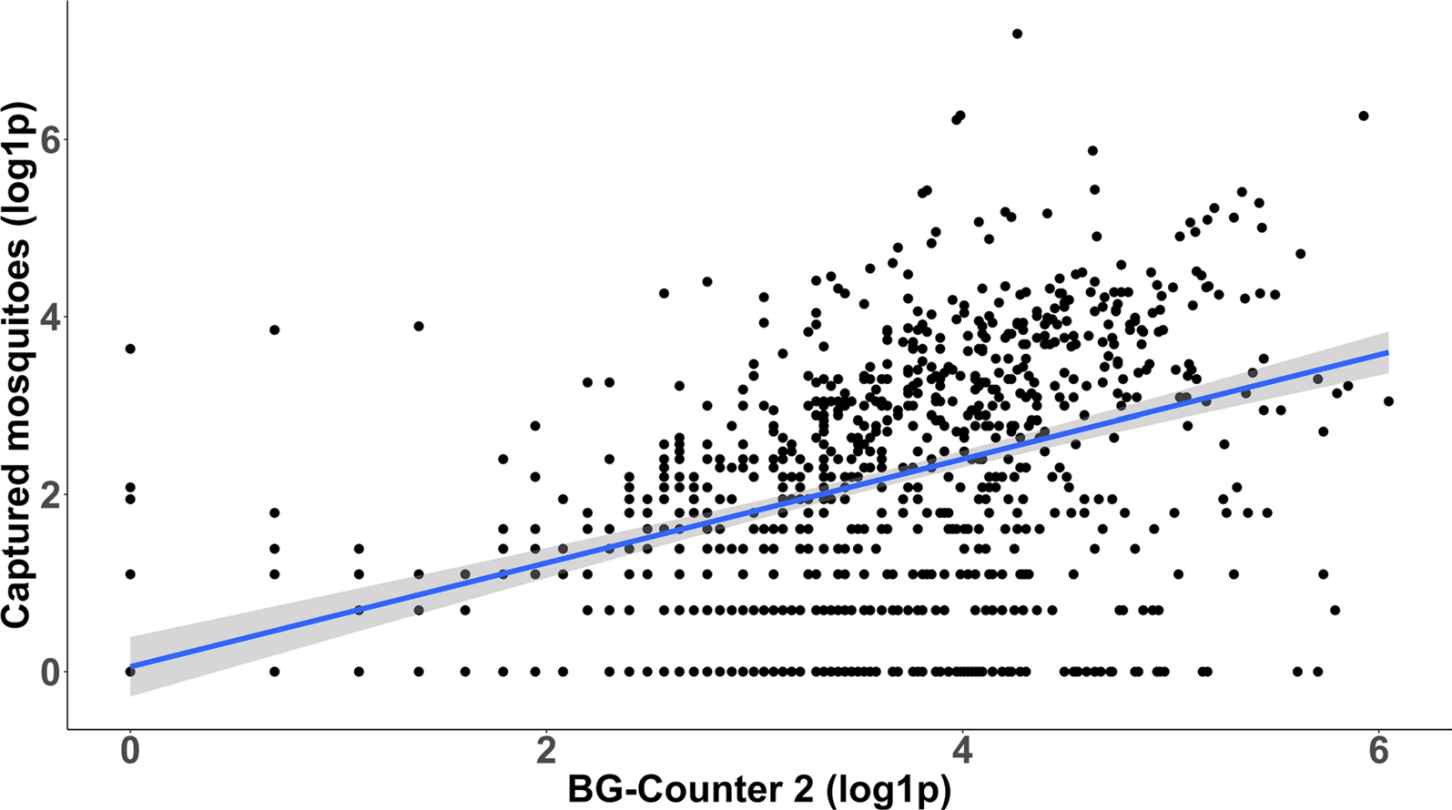
Linear regression between the BG-Counter counts and actually captured mosquitoes. Shaded area indicates ±SE

A linear model for all available data with the number of captured mosquitoes as a predictor variable and counted (BG-Counter) mosquitoes as response variables was significant (*R*^2^ = 0.16, *F*_(1, 860)_ = 165.0, *P* < 0.0001; Fig. 4). For all 18 trap sites with 10 or more trap days, 12 trap sites showed a significant, positive linear relationship between identified and counted mosquitoes, i.e. six trap sites equipped with the CO_2_-Pro and six trap sites equipped with the CO_2_-Pro-gravid (Table 2). There were no significant differences in the *R*^2^ values between the two trap versions (CO_2_-Pro: mean = 0.29, 95% CI = 0.1–0.5; CO_2_-Pro-gravid: 0.17, 95% CI = 0.1–03; Kruskal–Wallis, Chi-square = 1.2, *df* = 1, *P* = 0.270; both traps: mean = 0.23 95% CI = 0.14–0.32; Table 2).

**Table 2:** Summary statistics per sampling site with information on the results of linear models (lm) and Spearman correlations between the BG-Counter counts and actually captured mosquitoes for sampling sites with more than 10 trap days

Location	Trap	Trap days	Captured mosquitoes	Mean captured mosquitoes	lm: *R*-squared	lm: *P*-value	lm: Estimate	Accuracy (%)	Spearman correlation	Cor: *P*-value
Buchholz	CO_2_-Pro	14	245	17.5	0.58	0.0015	0.58	44.1	0.78	0.0010
Fürth	CO_2_-Pro-gravid	74	1233	16.7	0.16	0.0005	0.27	14.1	0.45	< 0.0001
Großheide	CO_2_-Pro	12	171	14.3	0.04	0.5426	0.09	20.1	0.18	0.5850
Hamburg 1	CO_2_-Pro-gravid	110	3918	35.6	0.20	< 0.0001	0.20	41.7	0.49	< 0.0001
Hamburg 1	CO_2_-Pro	104	1020	9.8	0.16	< 0.0001	0.25	36.8	0.50	< 0.0001
Hamburg 2	CO_2_-Pro-gravid	33	1373	41.6	0.36	0.0002	0.21	40.0	0.58	0.0004
Hamburg 2	CO_2_-Pro	34	250	7.4	0.34	0.0003	0.33	39.2	0.53	0.0013
Hamburg 3	CO_2_-Pro	36	530	14.7	0.48	< 0.0001	0.62	52.6	0.83	< 0.0001
Landau	CO_2_-Pro-gravid	23	179	7.8	0.04	0.3787	0.11	12.2	0.26	0.2330
Landau	CO_2_-Pro	22	568	25.8	0.62	< 0.0001	0.36	26.6	0.66	0.0009
March	CO_2_-Pro-gravid	19	234	12.3	0.22	0.0443	0.21	23.3	0.50	0.0309
Neu Wulmsdorf	CO_2_-Pro-gravid	19	1418	74.6	0.20	0.0551	0.23	47.3	0.39	0.0978
Neu Wulmsdorf	CO_2_-Pro	11	550	50	0.10	0.3518	0.21	37.0	0.55	0.0816
Tübingen	CO_2_-Pro-gravid	60	1471	24.5	0.21	0.0003	0.28	32.6	0.55	< 0.0001
Tübingen	CO_2_-Pro	50	302	6.0	0.07	0.0683	0.27	14.5	0.25	0.0766
Varel 1	CO_2_-Pro-gravid	33	1031	31.2	0.03	0.3061	0.08	33.7	0.10	0.5860
Weinheim	CO_2_-Pro-gravid	80	2504	31.3	0.15	0.0005	0.42	35.9	0.26	0.0184
Weinheim	CO_2_-Pro	78	1040	13.3	0.27	< 0.0001	0.34	27.1	0.48	< 0.0001
Mean (95% confidence interval)		45.1 (29.3–60.9)	1002.1 (526.5–1477.6)		0.2 (0.2–0.3)			32.2 (26.3–38.1)	0.5 (0.4–0.6)	

**Fig. 5:**
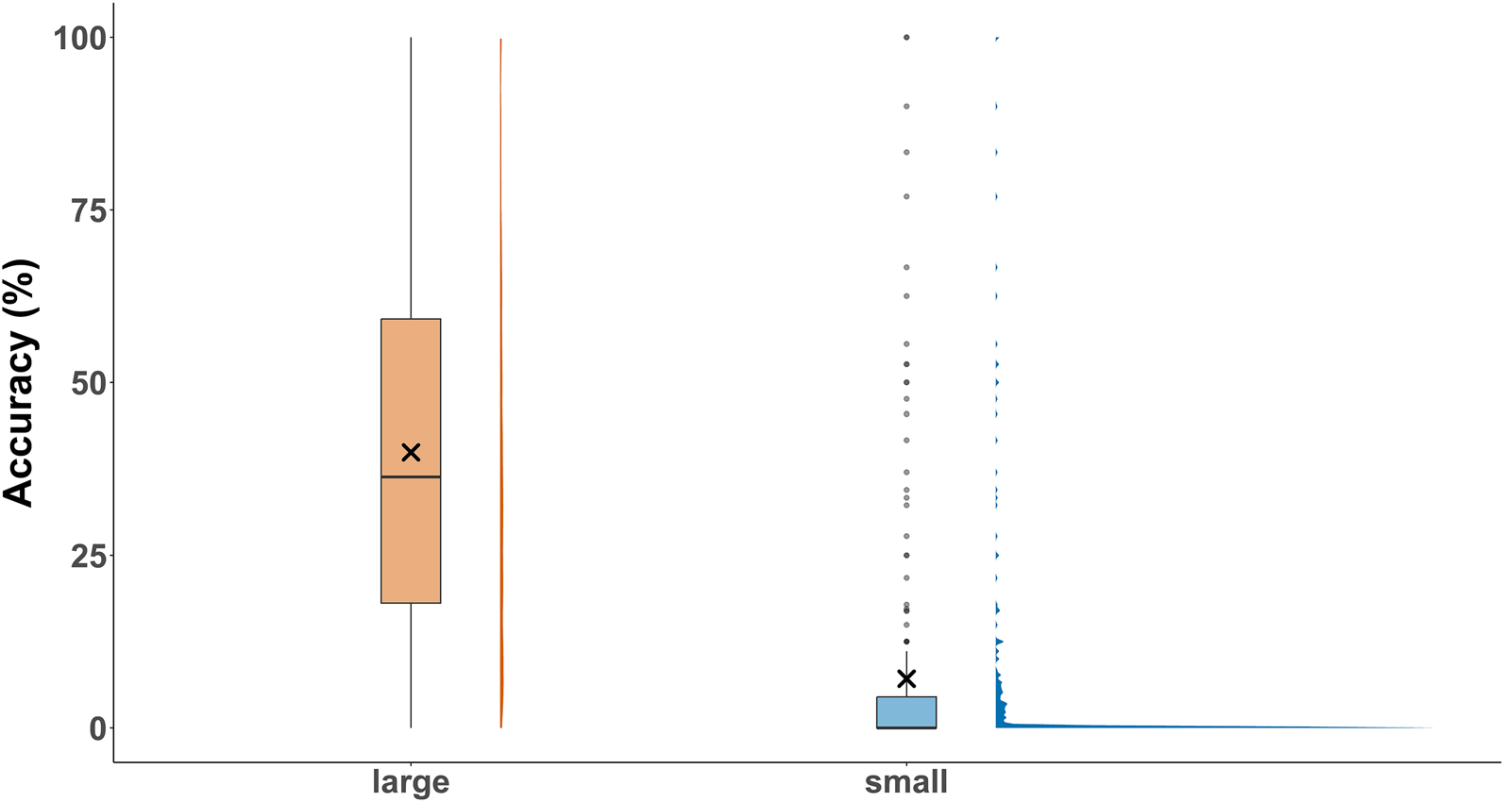
Boxplots comparing the BG-Counter accuracy (%) for large sample sizes (> 10 captured mosquitoes per day) and small sample sizes (< 10 captured mosquitoes per day) and half-density distribution. The X indicates the mean accuracy

**Fig. 6:**
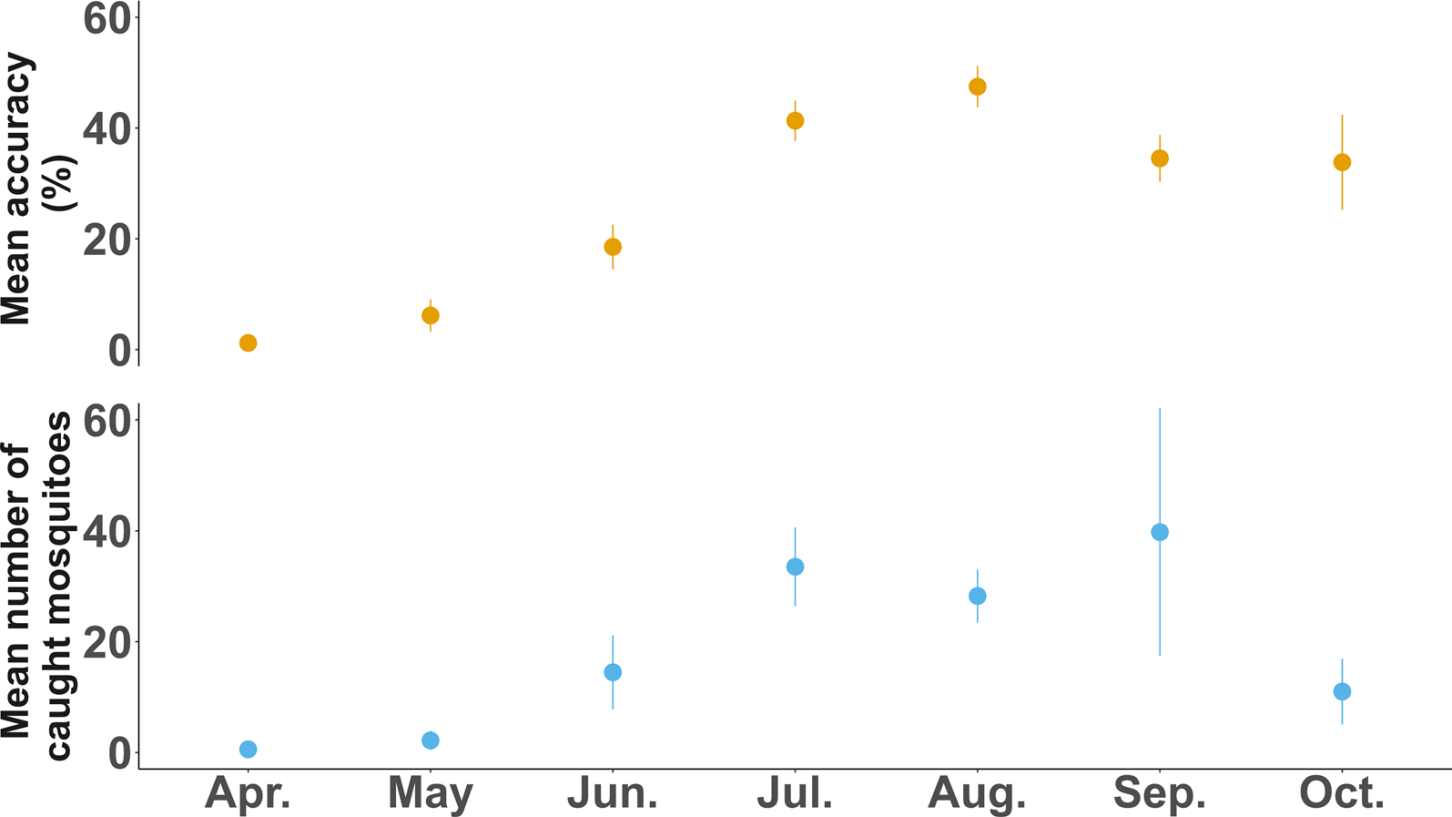
Mean accuracy (%) and mean captured mosquitoes per month ±CI

**Fig. 7:**
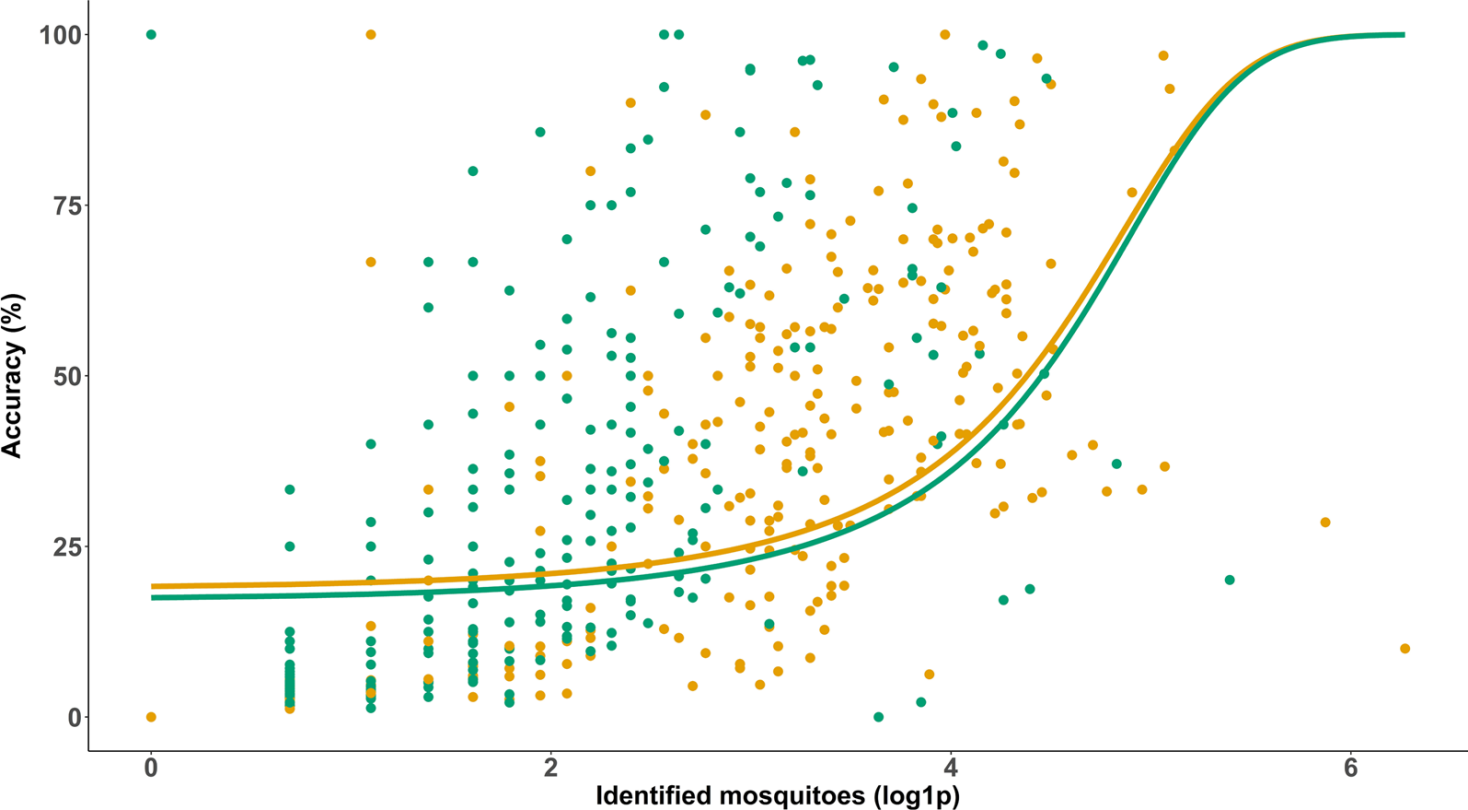
Binomial generalized linear mixed model between the BG-Counter accuracy (%) and captured mosquitoes separated by trap version (green: CO2-Pro, yellow: CO2-Pro-gravid)

The mean counting accuracy of the BG-Counter differed significantly between the CO_2_-Pro trap (mean accuracy = 29.5, 95% CI = 26.2–32.7) and the CO_2_-Pro-gravid trap (mean accuracy = 36.6, 95% CI = 33.4–39.7; Kruskal–Wallis, Chi-square = 11.9, *df* = 1, *P* = 0.0006; Fig. 3D). The mosquito sample size has a significant effect on the accuracy of the BG-Counter (Fig. 5). While captures with a small sample size (0–10 mosquitoes/24 h) had mean accuracy of 7.1% (95% CI = 4.8–9.4%), captures with a large sample size (> 10 mosquitoes/24 h) had mean accuracy of 39.9% (95% CI = 37.9–42.0%) (Kruskal–Wallis, Chi-square = 335.9, *df* = 1, *P* < 0.0001). The effect of the sample size also at least partly explains the differences in the mean accuracy between the sampling months (Fig. 6). It starts low in April (1.2%) and constantly increases, reaching its peak in August (47.5%). The daily accuracy and the month of capture were significantly correlated (GLMM binomial, *z* = 6.98, *P* < 0.0001). A joint analysis of both variables indicated that the number of collected mosquito specimens had a significant impact on the accuracy (GLMM binomial, *z* = 4.03, *P* < 0.0001; Fig. 7), while the trap version had no significant impact (GLMM binomial, *z* = −0.21, *P* = 0.83; Fig. 7).

## Discussion

With the invention of smart automatic traps for mosquitoes, a milestone in vector control and surveillance is met, which can revolutionize our ability to monitor mosquito populations in near real-time and enable a more targeted risk assessment and effective control measurement. However, our study highlights different things, which have to be kept in mind when setting up automatic mosquito traps and interpreting the collected data. The linear model with the captured mosquitoes as response and the counted specimens as predictor only explained little of the variation within the data (*R*^2^ = 0.16), but per individual trap the value can reach up to 0.62 (mean *R*^2^ = 0.23). As already emphasized by Day et al. [15], this very low accuracy demonstrates that the BG-Counter does not accurately count the absolute number of collected mosquitoes. However, the *R*^2^-value per individual trap shows that the information from the BG-Counter informs us in a predictable manner about the local increase and decrease in mosquito abundance, such as for the analysis of phenological patterns. The accuracy of the traps (12–53%) was lower than that presented in previous studies: 9.4–80.1% [15] and 62.3–98.7% [33]. The lower number of collected mosquito specimens (mean = 23.5) in our study likely explains the lower accuracy relative to previous studies with much higher numbers: mean = 678.8 [15] and mean = 151.4 [33]. This effect is not so pronounced in the prototype by Gonzales-Perez et al. [10], since the daily average mosquitoes caught was 61.2 and the overall average accuracy was 88.1. However, a slight trend can be observed for an increase in accuracy with mosquito abundance. For the BG-Counter, this relationship can also be observed for the mean number of mosquitoes caught and counting accuracy per month, where the accuracy was highest during the months of high mosquito abundance, i.e. July–September. In addition, another explanation for the low accuracy in the beginning of the season could be the high abundance of misclassified non-target organisms during springtime, which were not analysed in this study. However, this would also not explain the decreasing accuracy during autumn. The significant correlation of the BG-Counter accuracy with the sample size also results in site-specific dependence of the BG-Counter accuracy in our dataset. Large samples generally have higher counting accuracy, resulting in higher accuracy for sampling sites with an overall higher mosquito abundance. This was previously also observed in North Carolina, where the three sites with the highest mean accuracy were those with the highest numbers of daily mean captures [15].

The modification of the CO_2_-Pro trap to a combination of CO_2_ and gravid trap was very effective. In line with previous studies comparing gravid traps with CO_2_-baited traps, the capture rates of gravid *Culex* females with the CO_2_-gravid traps were significantly higher than those for the standard CO_2_-only version [34, 35]. Moreover, the CO_2_-Pro-gravid also attracted more non-gravid female mosquitoes. Downward suction of a trap as we have done for the CO_2_-Pro-gravid has rather been shown to decrease the success in collecting mosquitoes [36]. However, greater sampling success was also observed when combining gravid traps with lures for host-seeking mosquitoes [37]. Therefore, although the exact reasons are unclear, the container with hay infusion is probably the reason for the increase in host-seeking mosquitoes for the gravid CO_2_-Pro-gravid. We found no statistically significant difference in the counting accuracy of the BG-Counter between the two trap versions CO_2_-Pro and CO_2_-Pro-gravid. For epidemiological studies in particular, non-nulliparous females are of utmost importance, since these have a higher probability of being infected with pathogens, but are only rarely captured by standard CO_2_-baited traps. Nevertheless, although not quantified in this study, the collection of a larger proportion of non-target species might be a disadvantage of the BG-Pro-gravid trap, as it could make sorting costlier. Non-target insects searching for breeding sites or a water source are attracted by the container with hay infusion. Although the BG-Counter has shortcomings, it is able to depict the relative phenology of mosquitoes. If absolute numbers are required, it is recommended that traps be placed at locations known for high mosquito abundance during such periods of high abundance. The performance of the BG-Counter might be further improved by increased CO_2_ levels, additional attractants and a trap design capturing multiple mosquito life stages (e.g. CO_2_-Pro-gravid). For use in settings with low mosquito abundance, the data should be interpreted only as relative abundance. Additionally, the possibility of increasing the counting accuracy post-capture should be explored, e.g. model-based seasonal or temperature-dependent error rates.

Finally, a few technical notes. The distance between the traps varied from sample site to sample site, but we placed the traps in similar surroundings, e.g. along a hedge. However, in general, strong differences can be expected for the number of captured mosquitoes and for traps in close proximity [38]. Unknown microclimatic differences such as wind speed, temperature and humidity have been shown to have large impacts on the capture of mosquitoes in different life stages [39]. The BG-Pro trap has a relatively strong fan and is therefore perceived as louder than other mosquito traps. The same applies to the sound of the magnet valve of the BG-Counter, which was often recognized as a nuisance. For an average of 15% of all sampling events, the BG-Counter did not manage to establish an Internet connection, resulting in captured mosquitoes without corresponding counter data.

## Conclusions

The CO_2_-Pro-gravid trap represents a useful modification of the standard CO_2_-Pro trap version, collecting a greater number of mosquitoes, particularly gravid *Culex* species. The accuracy of the BG-Counter was sufficient to capture the phenology of mosquitoes but varied considerably between the sampling sites and months. The counting accuracy seems to be strongly influenced by the number of mosquito specimens collected per sampling event and probably other confounding factors such as weather conditions or bycatch, which will require further research. This is the first large-scale performance assessment of an automatic mosquito counting device and can be used as a guideline, highlighting potential pitfalls for future study designs and interpretation of data. With the increasing development of Internet of Things (IoT) devices for the monitoring of mosquitoes, further independent research is needed in order to identify the advantages and disadvantages of such products.

### Supplementary Information

Supplementary Material 1. The figure shows the exposed sensor opening and the necessary modifications to mount the Counter in reverse orientation. The table displays the success rate of the Counter in transmitting measured data over the internet across all sampling sites.

## Data Availability

All data and a commented R Markdown file to reproduce the figures and statistical analysis are available under https://github.com/Silence1490/Large-scale-performance-assessment-of-the-BG-Counter-2-used-with-two-different-mosquito-traps/tree/main.
