# Supplementary material for: Large-scale performance assessment of the BG-Counter 2 used with two different mosquito traps
Source: Parasit Vectors. 2024 Jun 27;17:273. doi: 10.1186/s13071-024-06338-x (PMC11209956; doi:10.1186/s13071-024-06338-x)
Supplement: Supplementary file 1 — Supplementary Material 1. The figure shows the exposed sensor opening and the necessary modifications to mount the Counter in reverse orientation. The table displays the success rate of the Counter in transmitting measured data over the internet across all sampling sites. [file 13071_2024_6338_MOESM1_ESM.docx]

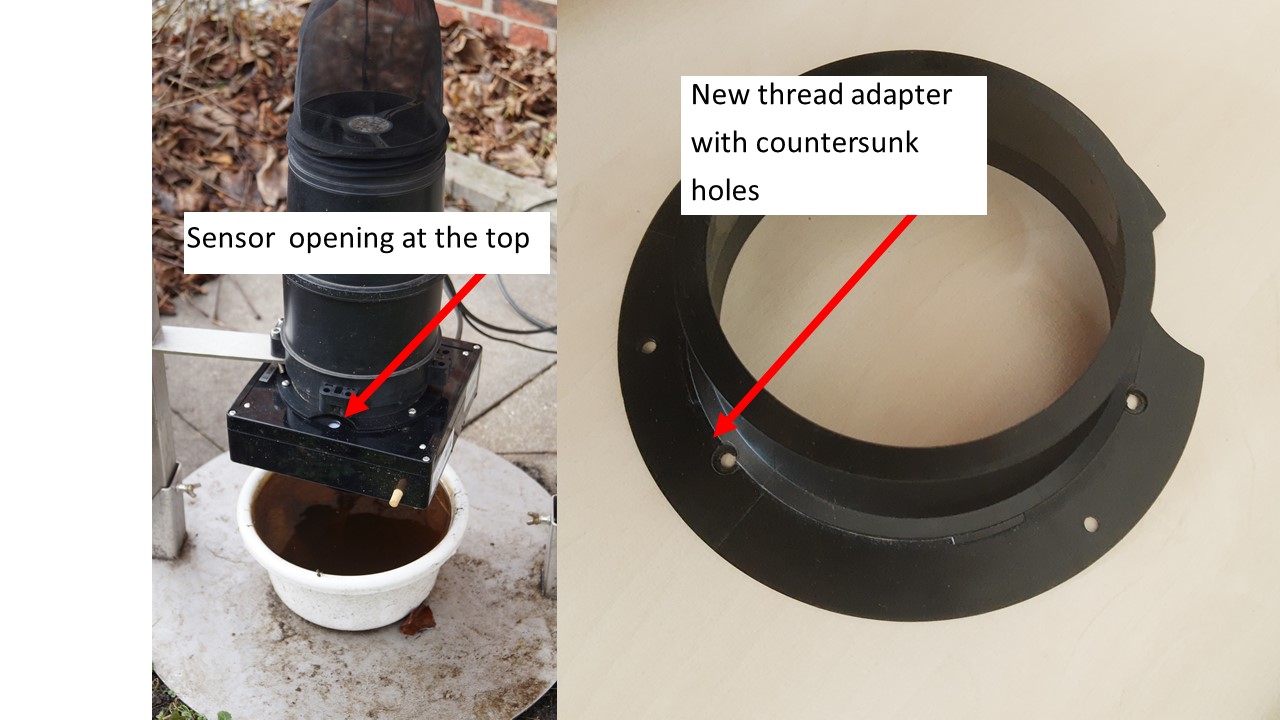


Figure S1: Exposed sensor opening (left) and altered thread adapter (right)

Table. S1: Transmission success rate of the BG-Counter

| Location | Trap | Days transmitted | Days captured | Transmission succcess |
| --- | --- | --- | --- | --- |
| Berlin | CO_2_-Pro-gravid | 9 | 9 | 100 |
| Buchholz | CO_2_-Pro-gravid | 9 | 11 | 81.8 |
| Buchholz | CO_2_-Pro | 14 | 15 | 93.3 |
| Düsseldorf | CO_2_-Pro-gravid | 3 | 3 | 100 |
| Friedberg | CO_2_-Pro-gravid | 5 | 5 | 100 |
| Fürth | CO_2_-Pro-gravid | 74 | 152 | 48.7 |
| Großheide | CO_2_-Pro-gravid | 7 | 7 | 100 |
| Großheide | CO_2_-Pro | 11 | 11 | 100 |
| Hamburg 1 | CO_2_-Pro-gravid | 109 | 109 | 100 |
| Hamburg 1 | CO_2_-Pro | 104 | 104 | 100 |
| Hamburg 2 | CO_2_-Pro-gravid | 32 | 32 | 100 |
| Hamburg 2 | CO_2_-Pro | 34 | 34 | 100 |
| Hamburg 3 | CO_2_-Pro | 36 | 62 | 58.1 |
| Konstanz | CO_2_-Pro-gravid | 5 | 7 | 71.4 |
| Landau | CO_2_-Pro-gravid | 23 | 23 | 100 |
| Landau | CO_2_-Pro | 22 | 22 | 100 |
| March | CO_2_-Pro-gravid | 19 | 19 | 100 |
| Neu Wulmsdorf | CO_2_-Pro-gravid | 18 | 18 | 100 |
| Neu Wulmsdorf | CO_2_-Pro | 11 | 13 | 84.6 |
| Regensburg | CO_2_-Pro-gravid | 5 | 11 | 45.5 |
| Tübingen | CO_2_-Pro-gravid | 60 | 71 | 84.5 |
| Tübingen | CO_2_-Pro | 50 | 50 | 100 |
| Varel 1 | CO_2_-Pro-gravid | 33 | 33 | 100 |
| Varel 2 | CO_2_-Pro-gravid | 3 | 17 | 17.6 |
| Weinheim | CO_2_-Pro-gravid | 78 | 78 | 100 |
| Weinheim | CO_2_-Pro | 78 | 78 | 100 |
| Wittenberg | CO_2_-Pro | 2 | 6 | 33.3 |
| Mean |  | 31.6  (CI:19 - 44.2) | 37  (CI:21.8 - 52.3) | 85.9  (CI:76.4 - 95.4) |
